# Bis(dimethyl sulfoxide-κ*O*)bis(saccharinato-κ*N*)­zinc(II)

**DOI:** 10.1107/S1600536811045703

**Published:** 2011-11-05

**Authors:** Fezile S. W. Potwana, Bongakonke E. Shandu, Werner E. Van Zyl

**Affiliations:** a School of Chemistry, University of KwaZulu-Natal, Westville Campus, Private Bag X54001, Durban 4000, South Africa

## Abstract

The title compound, [Zn(C_7_H_4_N_2_O_3_S)_2_(C_2_H_6_OS)_2_], is a neutral four-coordinate complex with a tetra­hedral geometry. The metal atom is surrounded by the two dimethyl sulfoxide (DMSO) ligands, each coordinating through the O atom, and two anionic saccharinate (1,1,3-trioxo-2,3-dihydro-1λ^6^,2-benzo­thia­zol-2-ide) ligands coordinating through the N atom. The tetra­hedral geometry is slightly distorted as is evident from the N—Zn—N bond angle of 113.85 (6)°, the O—Zn—O bond angle of 98.92 (6)° and O—Zn—N bond angles of 116.96 (6) and 103.93 (6)°. The Zn—N bond lengths are 1.9742 (15) and 2.0025 (16) Å. The Zn—O bond lengths are 1.9806 (14) Å and 1.9468 (14) Å. The DMSO ligand coordinates through the lone pair of electrons on the O atom, as can be seen from the Zn—O—S bond angle of 131.30 (8)°.

## Related literature

For a general review article on the coordination chemistry of saccharinate ligands, see: Baran & Yilmaz (2006 ▶). For a zinc(II) complex with saccharinate as a polyfunctional ligand, see: Yilmaz *et al.* (2006 ▶) and for zinc(II) complexes with saccharinate as a non-coordinating ligand, see: Batsanov *et al.* (2011 ▶). For the general preparation of saccharinate precursor complexes, see: Haider *et al.* (1985 ▶).
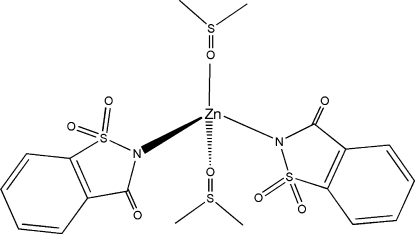


## Experimental

### 

#### Crystal data

[Zn(C_7_H_4_N_2_O_3_S)_2_(C_2_H_6_OS)_2_]
*M* *_r_* = 585.97
Monoclinic, 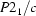

*a* = 19.2506 (7) Å
*b* = 8.2855 (3) Å
*c* = 14.8880 (5) Å
β = 103.460 (1)°
*V* = 2309.42 (14) Å^3^
*Z* = 4
Mo *K*α radiation
μ = 1.47 mm^−1^
*T* = 173 K
0.14 × 0.11 × 0.05 mm

#### Data collection

Bruker Kappa DUO APEXII diffractometer
Absorption correction: multi-scan (*SADABS*; Sheldrick, 1997 ▶) *T*_min_ = 0.820, *T*_max_ = 0.930
44984 measured reflections
5730 independent reflections
4739 reflections with *I* > 2σ(*I*)
*R*_int_ = 0.047

#### Refinement

*R*[*F* ^2^ > 2σ(*F* ^2^)] = 0.027
*wR*(*F* ^2^) = 0.067
*S* = 1.02
5730 reflections
302 parameters
H-atom parameters constrained
Δρ_max_ = 0.42 e Å^−3^
Δρ_min_ = −0.30 e Å^−3^

### 

Data collection: *APEX2* (Bruker, 2006 ▶); cell refinement: *SAINT* (Bruker, 2006 ▶); data reduction: *SAINT*; program(s) used to solve structure: *SHELXS97* (Sheldrick, 2008 ▶); program(s) used to refine structure: *SHELXL97* (Sheldrick, 2008 ▶); molecular graphics: *SHELXTL* (Sheldrick, 2008 ▶); software used to prepare material for publication: *SHELXL97*.

## Supplementary Material

Crystal structure: contains datablock(s) I, global. DOI: 10.1107/S1600536811045703/fj2470sup1.cif

Structure factors: contains datablock(s) I. DOI: 10.1107/S1600536811045703/fj2470Isup2.hkl

Additional supplementary materials:  crystallographic information; 3D view; checkCIF report

## Figures and Tables

**Fig. 1.**
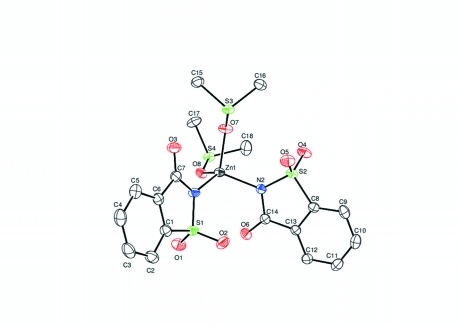
Molecular structure (ORTEP) of the title complex drawn at 50% ellipsoid probablility.

## supplementary crystallographic information

### Comment

Saccharin (*o*-sulfobenzimide; 1,2-benzothiazole-3(2*H*)-one 1,1-dioxide; Hsac) is a widely used artificial sweetening agent. The imino hydrogen is acidic and can be readily deprotonated. The coordination chemistry of this anion is versatile due to the different coordination sites to metallic centers it can accommodate, *i.e.*, one N, one O (carbonylic) and two O (sulfonic) atoms. These donor atoms of the anion can thus readily generate either N– or O-monodentate or bidentate (N, O) coordination. Saccharin is normally used as the sodium or calcium salt which dramatically improves water solubility. Most metal complexes contain the deprotonated form of saccharin, and this saccharinate anion (sac) is commercially available as the sodium salt, used in the present study. The reaction of sodium saccharinate with a variety of divalent transition metal ions results in coordination complexes with general formula [*M*(sac)_2_(H_2_O)_4_].2H_2_O, (*M* = V, Cr, Mn, Fe, Co, Ni, Cu, Zn, Cd), which all show a clear preference to bind through the deprotonated anionic N-atom (Baran and Yilmaz, 2006). These octahedral complexes contain two N-bonded sac ligands in *trans* positions, and complexes of the type [*M*(sac)_2_(H_2_O)_4_].2H_2_O are thus commonly used as precursors in the synthesis of mixed-ligand saccharinate complexes. The aqua ligands in these metal complexes are labile and readily displaced by direct reaction of neutral ligands. The addition of strongly donating ligands to the solutions of the complexes usually results in the substitution of all four aqua ligands, thereby forming stable new mixed-ligand complexes. In cases where the incoming neutral ligand is relatively bulky, as in the present study, it causes steric hindrance and once all four aqua ligands become displaced, the Zn center adopts a tetrahedral geometry, rather than octahedral. Although there are a number of Zn(II) saccharinate complexes previously reported (Batsanov *et al.*, 2011, and refs. therein), we are unaware of any report where both saccharinate and DMSO ligands are present in a structurally characterized Zn(II) complex.

### Experimental

[Zn(sac)_2_(H_2_O)_4_].2H_2_O was prepared as per literature method (Haider *et al.*, 1985). Colorless crystals of [Zn(sac)_2_(H_2_O)_4_].2H_2_O (1.60 g; 2.82 mmol) was placed in a 100 ml beaker and dissolved in excess amount of dimethyl sulfoxide (DMSO) (20 ml). The reaction mixture was gently heated on a heating mantle with stirring to reduce the volume of DMSO to ~7 ml. The beaker was removed from the heat source and allowed to stand for 6 days during which time large colorless blocky crystals of the title compound were obtained. Yield (1.51 g, 92%); Mp 190°C; ^13^C NMR (CD_3_OD, 101 MHz) δ(p.p.m.): 40.37 (CH_3_-DMSO), 121.23 (C_6_-ring), 124.89 (C_6_-ring), 133.32 (C_6_-ring), 134.21 (C_6_-ring), 134.27 (C_6_-ring), 144.80 (C_6_-ring) 171.57 (C=O); IR (ATR) 1687 ν(C=O), 1596, 1419 ν(C=C), 1274, 1245 ν(O=S=O); 1138, 955 ν(S=O).

### Refinement

All non-hydrogen atoms were refined anisotropically. All hydrogen atoms could be found in the difference electron density maps and but were placed in idealized positions refining in riding models with *U*_iso_ set at 1.2 or 1.5 times those of their parent atoms and bond length of C—H ranging from 0.95 Å to 0.98 Å. The structure was refined to *R* factor of 0.0269.

### Crystal data

**Table:**

[Zn(C_7_H_4_N_2_O_3_S)_2_(C_2_H_6_OS)_2_]	*F*(000) = 1200
*M**_r_* = 585.97	*D*_x_ = 1.685 Mg m^−^^3^
Monoclinic, *P*2_1_/*c*	Mo *K*α radiation, λ = 0.71073 Å
Hall symbol: -P 2ybc	Cell parameters from 44984 reflections
*a* = 19.2506 (7) Å	θ = 2.2–28.4°
*b* = 8.2855 (3) Å	µ = 1.47 mm^−^^1^
*c* = 14.8880 (5) Å	*T* = 173 K
β = 103.460 (1)°	Plate, colourless
*V* = 2309.42 (14) Å^3^	0.14 × 0.11 × 0.05 mm
*Z* = 4	

### Data collection

**Table:**

Bruker Kappa DUO APEXII diffractometer	5730 independent reflections
Radiation source: fine-focus sealed tube	4739 reflections with *I* > 2σ(*I*)
graphite	*R*_int_ = 0.047
0.5° φ scans and ω scans	θ_max_ = 28.4°, θ_min_ = 2.2°
Absorption correction: multi-scan (*SADABS*; Sheldrick, 1997)	*h* = −25→25
*T*_min_ = 0.820, *T*_max_ = 0.930	*k* = −11→11
44984 measured reflections	*l* = −19→19

### Refinement

**Table:**

Refinement on *F*^2^	Primary atom site location: structure-invariant direct methods
Least-squares matrix: full	Secondary atom site location: difference Fourier map
*R*[*F*^2^ > 2σ(*F*^2^)] = 0.027	Hydrogen site location: inferred from neighbouring sites
*wR*(*F*^2^) = 0.067	H-atom parameters constrained
*S* = 1.02	*w* = 1/[σ^2^(*F*_o_^2^) + (0.0289*P*)^2^ + 1.4252*P*] where *P* = (*F*_o_^2^ + 2*F*_c_^2^)/3
5730 reflections	(Δ/σ)_max_ = 0.001
302 parameters	Δρ_max_ = 0.42 e Å^−^^3^
0 restraints	Δρ_min_ = −0.30 e Å^−^^3^

### Special details

**Table:**

Geometry. All e.s.d.'s (except the e.s.d. in the dihedral angle between two l.s. planes) are estimated using the full covariance matrix. The cell e.s.d.'s are taken into account individually in the estimation of e.s.d.'s in distances, angles and torsion angles; correlations between e.s.d.'s in cell parameters are only used when they are defined by crystal symmetry. An approximate (isotropic) treatment of cell e.s.d.'s is used for estimating e.s.d.'s involving l.s. planes.
Refinement. Refinement of *F*^2^ against ALL reflections. The weighted *R*-factor *wR* and goodness of fit *S* are based on *F*^2^, conventional *R*-factors *R* are based on *F*, with *F* set to zero for negative *F*^2^. The threshold expression of *F*^2^ > σ(*F*^2^) is used only for calculating *R*-factors(gt) *etc*. and is not relevant to the choice of reflections for refinement. *R*-factors based on *F*^2^ are statistically about twice as large as those based on *F*, and *R*- factors based on ALL data will be even larger.

### Fractional atomic coordinates and isotropic or equivalent isotropic displacement parameters (Å^2^)

**Table:**

	*x*	*y*	*z*	*U*_iso_*/*U*_eq_	
Zn1	0.262359 (11)	0.60399 (3)	0.400931 (15)	0.01948 (6)	
S1	0.35238 (2)	0.41221 (6)	0.57598 (3)	0.02170 (10)	
S2	0.11081 (2)	0.42199 (6)	0.34334 (3)	0.02120 (10)	
S3	0.26721 (3)	0.48184 (6)	0.20501 (3)	0.02117 (10)	
S4	0.22966 (3)	0.98355 (6)	0.38273 (3)	0.02200 (10)	
O1	0.36505 (8)	0.54605 (18)	0.63895 (10)	0.0320 (3)	
O2	0.29500 (8)	0.30549 (18)	0.58307 (10)	0.0311 (3)	
O3	0.40031 (8)	0.44123 (19)	0.35058 (10)	0.0315 (3)	
O4	0.06265 (8)	0.52127 (18)	0.27742 (10)	0.0320 (3)	
O5	0.14742 (8)	0.29900 (17)	0.30376 (10)	0.0303 (3)	
O6	0.18678 (8)	0.60520 (18)	0.56992 (10)	0.0289 (3)	
O7	0.24945 (8)	0.61720 (16)	0.26750 (9)	0.0263 (3)	
O8	0.27128 (7)	0.83728 (16)	0.43039 (9)	0.0236 (3)	
N1	0.34324 (8)	0.47292 (19)	0.46907 (11)	0.0213 (3)	
N2	0.16708 (8)	0.53038 (19)	0.41761 (11)	0.0213 (3)	
C1	0.43194 (10)	0.3045 (2)	0.58191 (14)	0.0239 (4)	
C2	0.47356 (11)	0.2218 (3)	0.65600 (15)	0.0307 (5)	
H2	0.4602	0.2140	0.7134	0.037*	
C3	0.53551 (12)	0.1510 (3)	0.64251 (17)	0.0355 (5)	
H3	0.5656	0.0933	0.6917	0.043*	
C4	0.55447 (11)	0.1632 (3)	0.55787 (17)	0.0343 (5)	
H4	0.5972	0.1134	0.5503	0.041*	
C5	0.51199 (10)	0.2470 (2)	0.48461 (16)	0.0286 (4)	
H5	0.5251	0.2552	0.4271	0.034*	
C6	0.45011 (10)	0.3180 (2)	0.49758 (14)	0.0224 (4)	
C7	0.39693 (10)	0.4160 (2)	0.42994 (14)	0.0226 (4)	
C8	0.06604 (10)	0.3411 (2)	0.42394 (13)	0.0208 (4)	
C9	0.00967 (10)	0.2333 (2)	0.41005 (15)	0.0280 (4)	
H9	−0.0105	0.1886	0.3510	0.034*	
C10	−0.01597 (11)	0.1938 (2)	0.48726 (16)	0.0306 (5)	
H10	−0.0546	0.1201	0.4808	0.037*	
C11	0.01342 (11)	0.2592 (3)	0.57333 (15)	0.0298 (5)	
H11	−0.0048	0.2282	0.6249	0.036*	
C12	0.06915 (10)	0.3695 (2)	0.58516 (14)	0.0252 (4)	
H12	0.0889	0.4156	0.6440	0.030*	
C13	0.09509 (9)	0.4103 (2)	0.50915 (13)	0.0196 (4)	
C14	0.15383 (10)	0.5259 (2)	0.50515 (13)	0.0208 (4)	
C15	0.33065 (11)	0.5740 (3)	0.15131 (14)	0.0284 (4)	
H15A	0.3128	0.6796	0.1267	0.043*	
H15B	0.3381	0.5052	0.1008	0.043*	
H15C	0.3760	0.5877	0.1969	0.043*	
C16	0.19112 (11)	0.4860 (3)	0.11028 (14)	0.0283 (4)	
H16A	0.1487	0.4530	0.1314	0.042*	
H16B	0.1985	0.4116	0.0622	0.042*	
H16C	0.1843	0.5957	0.0851	0.042*	
C17	0.26648 (13)	1.0286 (3)	0.28680 (16)	0.0345 (5)	
H17A	0.3152	1.0698	0.3089	0.052*	
H17B	0.2370	1.1104	0.2481	0.052*	
H17C	0.2675	0.9304	0.2504	0.052*	
C18	0.14491 (11)	0.9084 (3)	0.32281 (18)	0.0385 (6)	
H18A	0.1513	0.8331	0.2746	0.058*	
H18B	0.1146	0.9985	0.2945	0.058*	
H18C	0.1221	0.8524	0.3663	0.058*	

### Atomic displacement parameters (Å^2^)

**Table:**

	*U*^11^	*U*^22^	*U*^33^	*U*^12^	*U*^13^	*U*^23^
Zn1	0.02243 (11)	0.01899 (11)	0.01713 (11)	0.00229 (8)	0.00485 (8)	0.00187 (8)
S1	0.0249 (2)	0.0211 (2)	0.0195 (2)	0.00290 (18)	0.00603 (17)	0.00380 (18)
S2	0.0267 (2)	0.0198 (2)	0.0162 (2)	0.00174 (17)	0.00311 (17)	0.00012 (17)
S3	0.0283 (2)	0.0189 (2)	0.0171 (2)	0.00189 (17)	0.00686 (18)	0.00173 (17)
S4	0.0286 (2)	0.0183 (2)	0.0203 (2)	0.00216 (18)	0.00797 (18)	0.00039 (18)
O1	0.0395 (8)	0.0306 (8)	0.0257 (8)	0.0059 (6)	0.0072 (6)	−0.0040 (6)
O2	0.0297 (7)	0.0308 (8)	0.0356 (9)	0.0000 (6)	0.0130 (6)	0.0106 (7)
O3	0.0321 (8)	0.0423 (9)	0.0211 (7)	0.0063 (7)	0.0084 (6)	0.0047 (6)
O4	0.0386 (8)	0.0332 (8)	0.0202 (7)	0.0066 (7)	−0.0010 (6)	0.0061 (6)
O5	0.0415 (8)	0.0250 (7)	0.0265 (8)	0.0044 (6)	0.0121 (6)	−0.0041 (6)
O6	0.0295 (7)	0.0319 (8)	0.0240 (7)	−0.0069 (6)	0.0037 (6)	−0.0076 (6)
O7	0.0401 (8)	0.0228 (7)	0.0168 (7)	0.0076 (6)	0.0078 (6)	0.0010 (6)
O8	0.0278 (7)	0.0202 (6)	0.0204 (7)	0.0017 (5)	0.0011 (5)	0.0013 (5)
N1	0.0238 (8)	0.0227 (8)	0.0178 (8)	0.0035 (6)	0.0053 (6)	0.0051 (6)
N2	0.0231 (8)	0.0222 (8)	0.0185 (8)	−0.0017 (6)	0.0043 (6)	−0.0001 (6)
C1	0.0243 (9)	0.0210 (9)	0.0247 (10)	0.0008 (7)	0.0021 (8)	0.0023 (8)
C2	0.0336 (11)	0.0295 (11)	0.0262 (11)	0.0014 (9)	0.0010 (9)	0.0053 (9)
C3	0.0290 (11)	0.0324 (11)	0.0383 (13)	0.0035 (9)	−0.0060 (9)	0.0074 (10)
C4	0.0228 (10)	0.0287 (11)	0.0491 (14)	0.0050 (8)	0.0035 (9)	0.0020 (10)
C5	0.0247 (10)	0.0263 (10)	0.0351 (12)	−0.0007 (8)	0.0077 (8)	−0.0023 (9)
C6	0.0219 (9)	0.0196 (9)	0.0244 (10)	−0.0007 (7)	0.0027 (7)	0.0005 (8)
C7	0.0231 (9)	0.0216 (9)	0.0234 (10)	−0.0004 (7)	0.0058 (7)	0.0003 (8)
C8	0.0235 (9)	0.0189 (9)	0.0192 (9)	0.0029 (7)	0.0031 (7)	0.0007 (7)
C9	0.0274 (10)	0.0224 (9)	0.0307 (11)	−0.0023 (8)	−0.0002 (8)	−0.0056 (8)
C10	0.0243 (10)	0.0236 (10)	0.0438 (13)	−0.0038 (8)	0.0076 (9)	0.0004 (9)
C11	0.0266 (10)	0.0311 (11)	0.0348 (12)	−0.0001 (8)	0.0132 (9)	0.0068 (9)
C12	0.0240 (9)	0.0301 (10)	0.0217 (10)	0.0000 (8)	0.0062 (7)	0.0004 (8)
C13	0.0193 (8)	0.0186 (8)	0.0200 (9)	0.0022 (7)	0.0030 (7)	0.0014 (7)
C14	0.0206 (9)	0.0195 (9)	0.0216 (9)	0.0018 (7)	0.0037 (7)	0.0007 (7)
C15	0.0348 (11)	0.0280 (10)	0.0242 (10)	−0.0046 (8)	0.0103 (8)	0.0030 (8)
C16	0.0312 (10)	0.0311 (11)	0.0208 (10)	0.0000 (8)	0.0022 (8)	0.0006 (8)
C17	0.0431 (13)	0.0335 (11)	0.0331 (12)	0.0056 (10)	0.0214 (10)	0.0122 (10)
C18	0.0265 (10)	0.0307 (11)	0.0544 (15)	0.0024 (9)	0.0017 (10)	0.0137 (11)

### Geometric parameters (Å, °)

**Table:**

Zn1—O7	1.9468 (14)	C4—C5	1.387 (3)
Zn1—N1	1.9742 (15)	C4—H4	0.9500
Zn1—O8	1.9806 (14)	C5—C6	1.382 (3)
Zn1—N2	2.0025 (16)	C5—H5	0.9500
S1—O1	1.4358 (15)	C6—C7	1.497 (3)
S1—O2	1.4379 (15)	C8—C9	1.383 (3)
S1—N1	1.6392 (16)	C8—C13	1.386 (3)
S1—C1	1.757 (2)	C9—C10	1.391 (3)
S2—O5	1.4407 (14)	C9—H9	0.9500
S2—O4	1.4408 (14)	C10—C11	1.385 (3)
S2—N2	1.6265 (16)	C10—H10	0.9500
S2—C8	1.765 (2)	C11—C12	1.389 (3)
S3—O7	1.5454 (14)	C11—H11	0.9500
S3—C15	1.780 (2)	C12—C13	1.381 (3)
S3—C16	1.782 (2)	C12—H12	0.9500
S4—O8	1.5328 (14)	C13—C14	1.494 (3)
S4—C17	1.776 (2)	C15—H15A	0.9800
S4—C18	1.780 (2)	C15—H15B	0.9800
O3—C7	1.216 (2)	C15—H15C	0.9800
O6—C14	1.216 (2)	C16—H16A	0.9800
N1—C7	1.382 (2)	C16—H16B	0.9800
N2—C14	1.385 (2)	C16—H16C	0.9800
C1—C6	1.384 (3)	C17—H17A	0.9800
C1—C2	1.384 (3)	C17—H17B	0.9800
C2—C3	1.385 (3)	C17—H17C	0.9800
C2—H2	0.9500	C18—H18A	0.9800
C3—C4	1.395 (3)	C18—H18B	0.9800
C3—H3	0.9500	C18—H18C	0.9800
			
O7—Zn1—N1	116.96 (6)	C1—C6—C7	112.12 (17)
O7—Zn1—O8	98.92 (6)	O3—C7—N1	124.39 (18)
N1—Zn1—O8	113.93 (6)	O3—C7—C6	124.33 (18)
O7—Zn1—N2	103.92 (6)	N1—C7—C6	111.28 (16)
N1—Zn1—N2	113.85 (6)	C9—C8—C13	122.64 (18)
O8—Zn1—N2	107.73 (6)	C9—C8—S2	129.34 (16)
O1—S1—O2	116.33 (10)	C13—C8—S2	107.96 (14)
O1—S1—N1	111.13 (9)	C8—C9—C10	116.43 (19)
O2—S1—N1	110.44 (9)	C8—C9—H9	121.8
O1—S1—C1	110.24 (9)	C10—C9—H9	121.8
O2—S1—C1	111.07 (9)	C11—C10—C9	121.70 (19)
N1—S1—C1	95.74 (9)	C11—C10—H10	119.2
O5—S2—O4	115.07 (9)	C9—C10—H10	119.1
O5—S2—N2	110.91 (9)	C10—C11—C12	120.8 (2)
O4—S2—N2	111.66 (9)	C10—C11—H11	119.6
O5—S2—C8	111.85 (9)	C12—C11—H11	119.6
O4—S2—C8	109.97 (9)	C13—C12—C11	118.23 (19)
N2—S2—C8	95.79 (9)	C13—C12—H12	120.9
O7—S3—C15	103.31 (9)	C11—C12—H12	120.9
O7—S3—C16	101.90 (9)	C12—C13—C8	120.20 (18)
C15—S3—C16	99.19 (10)	C12—C13—C14	127.67 (17)
O8—S4—C17	105.95 (9)	C8—C13—C14	112.14 (17)
O8—S4—C18	105.97 (9)	O6—C14—N2	123.72 (17)
C17—S4—C18	99.28 (12)	O6—C14—C13	125.08 (18)
S3—O7—Zn1	125.44 (8)	N2—C14—C13	111.20 (16)
S4—O8—Zn1	131.30 (8)	S3—C15—H15A	109.5
C7—N1—S1	112.50 (13)	S3—C15—H15B	109.5
C7—N1—Zn1	123.29 (13)	H15A—C15—H15B	109.5
S1—N1—Zn1	124.15 (9)	S3—C15—H15C	109.5
C14—N2—S2	112.67 (13)	H15A—C15—H15C	109.5
C14—N2—Zn1	119.96 (12)	H15B—C15—H15C	109.5
S2—N2—Zn1	124.62 (9)	S3—C16—H16A	109.5
C6—C1—C2	122.78 (19)	S3—C16—H16B	109.5
C6—C1—S1	108.33 (14)	H16A—C16—H16B	109.5
C2—C1—S1	128.86 (17)	S3—C16—H16C	109.5
C1—C2—C3	116.9 (2)	H16A—C16—H16C	109.5
C1—C2—H2	121.6	H16B—C16—H16C	109.5
C3—C2—H2	121.6	S4—C17—H17A	109.5
C2—C3—C4	121.0 (2)	S4—C17—H17B	109.5
C2—C3—H3	119.5	H17A—C17—H17B	109.5
C4—C3—H3	119.5	S4—C17—H17C	109.5
C5—C4—C3	121.1 (2)	H17A—C17—H17C	109.5
C5—C4—H4	119.5	H17B—C17—H17C	109.5
C3—C4—H4	119.5	S4—C18—H18A	109.5
C6—C5—C4	118.2 (2)	S4—C18—H18B	109.5
C6—C5—H5	120.9	H18A—C18—H18B	109.5
C4—C5—H5	120.9	S4—C18—H18C	109.5
C5—C6—C1	120.01 (18)	H18A—C18—H18C	109.5
C5—C6—C7	127.86 (19)	H18B—C18—H18C	109.5
			
C15—S3—O7—Zn1	−121.22 (11)	C1—C2—C3—C4	−0.1 (3)
C16—S3—O7—Zn1	136.21 (11)	C2—C3—C4—C5	0.2 (3)
N1—Zn1—O7—S3	32.67 (13)	C3—C4—C5—C6	−0.1 (3)
O8—Zn1—O7—S3	155.39 (10)	C4—C5—C6—C1	−0.1 (3)
N2—Zn1—O7—S3	−93.73 (11)	C4—C5—C6—C7	178.88 (19)
C17—S4—O8—Zn1	−80.10 (14)	C2—C1—C6—C5	0.2 (3)
C18—S4—O8—Zn1	24.75 (15)	S1—C1—C6—C5	178.41 (15)
O7—Zn1—O8—S4	42.42 (12)	C2—C1—C6—C7	−178.92 (18)
N1—Zn1—O8—S4	167.29 (10)	S1—C1—C6—C7	−0.7 (2)
N2—Zn1—O8—S4	−65.39 (12)	S1—N1—C7—O3	177.82 (17)
O1—S1—N1—C7	115.49 (14)	Zn1—N1—C7—O3	0.6 (3)
O2—S1—N1—C7	−113.84 (14)	S1—N1—C7—C6	−1.7 (2)
C1—S1—N1—C7	1.19 (15)	Zn1—N1—C7—C6	−178.98 (12)
O1—S1—N1—Zn1	−67.29 (13)	C5—C6—C7—O3	3.0 (3)
O2—S1—N1—Zn1	63.38 (13)	C1—C6—C7—O3	−178.00 (19)
C1—S1—N1—Zn1	178.40 (11)	C5—C6—C7—N1	−177.45 (19)
O7—Zn1—N1—C7	11.80 (17)	C1—C6—C7—N1	1.6 (2)
O8—Zn1—N1—C7	−102.78 (15)	O5—S2—C8—C9	−64.2 (2)
N2—Zn1—N1—C7	133.13 (14)	O4—S2—C8—C9	64.9 (2)
O7—Zn1—N1—S1	−165.12 (9)	N2—S2—C8—C9	−179.50 (18)
O8—Zn1—N1—S1	80.29 (12)	O5—S2—C8—C13	118.46 (14)
N2—Zn1—N1—S1	−43.79 (13)	O4—S2—C8—C13	−112.38 (14)
O5—S2—N2—C14	−120.91 (13)	N2—S2—C8—C13	3.18 (14)
O4—S2—N2—C14	109.32 (14)	C13—C8—C9—C10	−1.5 (3)
C8—S2—N2—C14	−4.86 (14)	S2—C8—C9—C10	−178.46 (15)
O5—S2—N2—Zn1	40.17 (13)	C8—C9—C10—C11	0.1 (3)
O4—S2—N2—Zn1	−89.60 (12)	C9—C10—C11—C12	1.2 (3)
C8—S2—N2—Zn1	156.22 (11)	C10—C11—C12—C13	−0.9 (3)
O7—Zn1—N2—C14	−172.33 (13)	C11—C12—C13—C8	−0.5 (3)
N1—Zn1—N2—C14	59.33 (15)	C11—C12—C13—C14	179.56 (18)
O8—Zn1—N2—C14	−68.04 (14)	C9—C8—C13—C12	1.7 (3)
O7—Zn1—N2—S2	27.87 (12)	S2—C8—C13—C12	179.26 (15)
N1—Zn1—N2—S2	−100.47 (11)	C9—C8—C13—C14	−178.29 (17)
O8—Zn1—N2—S2	132.16 (10)	S2—C8—C13—C14	−0.75 (19)
O1—S1—C1—C6	−115.29 (15)	S2—N2—C14—O6	−175.69 (16)
O2—S1—C1—C6	114.27 (14)	Zn1—N2—C14—O6	22.2 (2)
N1—S1—C1—C6	−0.25 (15)	S2—N2—C14—C13	5.08 (19)
O1—S1—C1—C2	62.8 (2)	Zn1—N2—C14—C13	−156.99 (12)
O2—S1—C1—C2	−67.6 (2)	C12—C13—C14—O6	−1.9 (3)
N1—S1—C1—C2	177.84 (19)	C8—C13—C14—O6	178.16 (18)
C6—C1—C2—C3	−0.1 (3)	C12—C13—C14—N2	177.37 (18)
S1—C1—C2—C3	−177.96 (17)	C8—C13—C14—N2	−2.6 (2)
